# Venous-derived angioblasts generate organ-specific vessels during zebrafish embryonic development

**DOI:** 10.1242/dev.129247

**Published:** 2015-12-15

**Authors:** Gideon Hen, Julian Nicenboim, Oded Mayseless, Lihee Asaf, Masahiro Shin, Giorgia Busolin, Roy Hofi, Gabriella Almog, Natascia Tiso, Nathan D. Lawson, Karina Yaniv

**Affiliations:** 1 Department of Biological Regulation, Weizmann Institute of Science, Rehovot 76100, Israel; 2 Program in Gene Function and Expression, University of Massachusetts Medical School, Worcester, MA 01605, USA; 3 Department of Biology, University of Padova, Padova I-35131, Italy

**Keywords:** Angioblast, Angiogenesis, Zebrafish

## Abstract

Formation and remodeling of vascular beds are complex processes orchestrated by multiple signaling pathways. Although it is well accepted that vessels of a particular organ display specific features that enable them to fulfill distinct functions, the embryonic origins of tissue-specific vessels and the molecular mechanisms regulating their formation are poorly understood. The subintestinal plexus of the zebrafish embryo comprises vessels that vascularize the gut, liver and pancreas and, as such, represents an ideal model in which to investigate the early steps of organ-specific vessel formation. Here, we show that both arterial and venous components of the subintestinal plexus originate from a pool of specialized angioblasts residing in the floor of the posterior cardinal vein (PCV). Using live imaging of zebrafish embryos, in combination with photoconvertable transgenic reporters, we demonstrate that these angioblasts undergo two phases of migration and differentiation. Initially, a subintestinal vein forms and expands ventrally through a Bone Morphogenetic Protein-dependent step of collective migration. Concomitantly, a Vascular Endothelial Growth Factor-dependent shift in the directionality of migration, coupled to the upregulation of arterial markers, is observed, which culminates with the generation of the supraintestinal artery. Together, our results establish the zebrafish subintestinal plexus as an advantageous model for the study of organ-specific vessel development and provide new insights into the molecular mechanisms controlling its formation. More broadly, our findings suggest that PCV-specialized angioblasts contribute not only to the formation of the early trunk vasculature, but also to the establishment of late-forming, tissue-specific vascular beds.

## Abstract (precis)

**Highlighted article:** A specialized pool of angioblasts is the origin of the zebrafish subintestinal plexus, a structure that gives rise to the organ-specific vessels of the gut, liver and pancreas.

## INTRODUCTION

Establishment of a functional vascular system is essential for proper tissue development. In vertebrates, this process involves the formation of the main axial vessels through vasculogenesis, followed by a step of sprouting angiogenesis, to generate the systemic vasculature (Adams and Alitalo, 2007). Later on, as development proceeds, additional vascular beds form in order to support the establishment, growth and proper functionality of different organs. At present, it is well accepted that vessels of a particular organ display specific features that enable them to fulfill distinct functions (Cleaver, 2004; Aird, 2007). Although a large bulk of data describing the development of the systemic vasculature has accumulated during the past decades (Potente et al., 2011), little is known about the embryonic origins and the molecular mechanisms underlying the formation of organ-specific vessels (Nolan et al., 2013).

Formation of the gastrointestinal (GI) tissues was shown to follow the establishment of the cardiovascular system. Therefore, organs develop in the presence of already-formed blood vessels and adjacent endothelial cells (ECs) (Lammert et al., 2003; Nikolova and Lammert, 2003). Signals derived from the endothelium are thought to establish the location, differentiation and morphology of the gastrointestinal (GI) organs. In turn, these organs drive adjacent ECs to acquire unique features in order to meet their specific needs (Nikolova and Lammert, 2003). That is the case, for instance, for liver sinusoidal ECs (LSECs), which possess fenestrae that can modify their size in response to different agents (Braet et al., 1995), or for β-cells in pancreatic islets, which are thought to secrete insulin into the bloodstream through endothelial fenestrae (Nikolova and Lammert, 2003). These examples clearly demonstrate the need for mutual feedback and interaction between the tissues and their surrounding endothelium, in order to generate a specific functional vasculature.

Previous reports analyzing the development of the zebrafish GI tract have described the intimate interaction between the organs and the vasculature in this system. At 24 hours postfertilization (hpf), endoderm-derived cells form the intestinal rod in the zebrafish midline (Field et al., 2003a). By 52 hpf, the alimentary canal, including the pharynx, esophagus, intestinal bulb (IB) and posterior intestine, is fully discernible (Field et al., 2003a; Wallace and Pack, 2003), and it is entirely wrapped by the subintestinal vein (SIV), supraintestinal artery (SIA) and interconnecting vessels (ICVs) at 5.5-7.5 days postfertilization (dpf) (Isogai et al., 2001). In the case of the liver, vascularization is thought to begin at 36 hpf, when the first ECs are found adjacent to the liver bud (Field et al., 2003a). By ∼60 hpf, hepatic vessels (HVs) have reached and penetrated the hepatocyte surfaces, populating the entire liver by 72 hpf (Korzh et al., 2008). In the mouse, liver development begins at embryonic day (E) 8.5-9, when cells from the endoderm give rise to the liver bud. Half a day later, ECs surround the liver endoderm and intermingle with delaminating liver cells (Matsumoto et al., 2001). In the rat, the deﬁnitive structural differentiation of LSECs was suggested to be achieved only in the perinatal period (Barberá-Guillem et al., 1986). Mature LSECs display the characteristic structure of unique capillaries, showing open pores or fenestrae, and lacking a diaphragm and a basal lamina underneath the endothelium (Braet and Wisse, 2002). The role of ECs in liver development is still under debate. In mouse embryos, for instance, ECs were shown to be necessary for hepatic formation (Matsumoto et al., 2001). In zebrafish, the results have remained controversial, with some reports showing that initial liver development takes place normally in embryos lacking a well-developed vasculature (Field et al., 2003a), whereas others claim that ECs are essential for polarization of hepatocytes and proper liver formation (Sakaguchi et al., 2008), as well as during late stages of liver morphogenesis (Korzh et al., 2008). Most recently, LSECs were shown to enhance hepatic regeneration and repair in the mouse through the release of angiocrine factors (Ding et al., 2010, 2014; Hu et al., 2014), highlighting the important clinical implications of tissue-specific vascular beds.

The development of the pancreas has been extensively studied both in mice and in zebrafish (Edlund, 2002; Murtaugh, 2007). In mouse embryos, ventral and dorsal pancreatic buds develop from the endoderm at E8.5-E9.5 and give rise to both endocrine and exocrine cells (Kim and Hebrok, 2001; Lammert et al., 2001). In the zebrafish, by contrast, the dorsal posterior bud gives rise only to endocrine cells, whereas the ventral anterior bud generates both exocrine and endocrine cells (Field et al., 2003b). The zebrafish pancreas was shown to form via fusion of posterior and anterior pancreatic buds at around 52 hpf (Field et al., 2003b). Although the posterior pancreatic bud already lies in contact with the main axial vessels at 24 hpf, the first ECs are detected throughout the developing pancreas only by ∼52 hpf (Field et al., 2003b). ECs were shown to be necessary for the formation of the pancreas in the mouse and in *Xenopus* (Lammert et al., 2001). Endothelium of the aorta, for instance, was shown to induce budding of the dorsal pancreatic endoderm, thereby promoting endocrine development (Lammert et al., 2001). In a similar fashion, only the ventral pancreatic bud adjacent to the endothelium of the right vitelline vein develops into pancreatic tissue, whereas the other bud regresses (Lammert et al., 2001). The central role of the endothelium in pancreatic development was further supported by findings demonstrating that EC-endoderm interactions are essential for expression of Pdx1 and insulin in isolated mouse embryonic tissues (Lammert et al., 2001) and promote dorsal pancreatic development via Ptf1a, which is required for pancreatic lineage specification (Yoshitomi and Zaret, 2004). Although these studies indicate a clear role for the endothelium during pancreatic development, early morphogenesis and differentiation of the zebrafish pancreas appear normal in embryos devoid of endothelial cells (Field et al., 2003b).

The subintestinal plexus of the developing zebrafish embryo comprises vessels that vascularize the gut, liver and pancreas and, as such, represents an ideal model to investigate the early steps of organ-specific vessel formation. This vascular bed develops on both sides of the yolk ball (Isogai et al., 2001) and includes the SIA, SIV and ICVs. In contrast to the intersegmental vessels (ISVs) of the trunk, which sprout following well-defined attracting and repulsive cues (Torres-Vázquez et al., 2004; Suli et al., 2006), the subintestinal plexus develops in the absence of any apparent tissue guiding its formation. Nonetheless, these vessels generate a highly stereotypical basket-shaped structure, which acquires its mature form at approximately 3 dpf (Isogai et al., 2001; Lenard et al., 2015). Although established as a model for the study of metabolic regulation of angiogenesis (Avraham-Davidi et al., 2012), drug evaluation (Serbedzija et al., 1999) and for assaying tumor angiogenesis (Nicoli and Presta, 2007), very little is known about the embryonic origins and molecular mechanisms regulating formation of the subintestinal plexus. The first anatomical description of the subintestinal plexus and its derivatives was obtained through the use of confocal micro-angiography (Isogai et al., 2001). Most recently, a report by Lenard et al. (2015) provided the first characterization of the morphological events underlying the formation of the subintestinal plexus and established it as a powerful model for the study of vessel pruning.

Here, we investigate the different steps underlying the embryonic development of the subintestinal plexus and provide a thorough characterization of its interactions with the nascent digestive system. We find that, in contrast to previously proposed models claiming contribution of the dorsal aorta (DA) to the formation of organ-specific vessels, both the arterial and the venous components of the zebrafish subintestinal plexus share a venous origin. We further use live imaging of transgenic zebrafish, in combination with lineage-tracing approaches, to characterize the sequence of events involved in shaping the subintestinal plexus and identify the molecular cues controlling each step. Together, our findings provide new insights into the origins and development of the gastrointestinal vasculature in the zebrafish embryo, establishing it as an advantageous model for the study of organ-specific vessel development.

## RESULTS

### Anatomical and molecular characterization of the subintestinal plexus

**Fig. 1.**
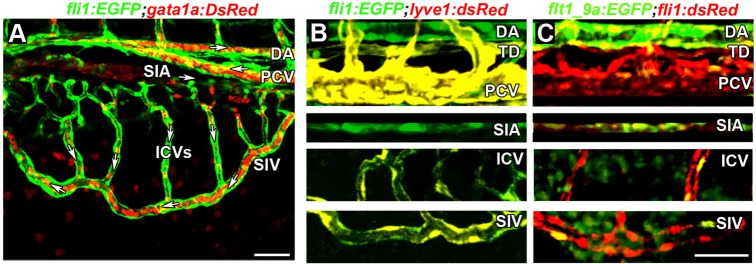
**Arterial-venous identity of the subintestinal vessels.** (A) Directionality of blood flow in the subintestinal plexus, as determined in *Tg(fli1:EGFP;gata1a:dsRed)* embryo at 3.5 dpf. White arrows indicate the direction of flow in the DA, PCV, SIA, SIV and ICVs. (B) Colocalization (yellow) of *fli1:EGFP* (green) and *lyve1:dsRed* fluorescence in venous ECs of the PCV, ICVs, TD and SIV of 4 dpf *Tg(fli1:EGFP;lyve1:dsRed)* double transgenic embryos. The DA and SIA show no *lyve1:dsRed* expression. (C) Colocalization (yellow) of *fli1*:*dsRed* and *flt1_9a* (green) in arterial ECs of the DA and SIA of 4 dpf *Tg(flt1_9a_cfos:GFP;fli1:dsRed)* double transgenic embryos. DA, dorsal aorta; ICVs, interconnecting vessels; PCV, posterior cardinal vein; SIA, supraintestinal artery; SIV, subintestinal vein; TD, thoracic duct. Scale bars: 50 µm.

Previous reports have provided a clear anatomical characterization of the subintestinal plexus in zebrafish and medaka (Isogai et al., 2001; Fujita et al., 2006) and have defined the identity of the different vessels composing this network, based on confocal microangiography analyses. Confocal imaging of 3.5 dpf *Tg(fli1:EGFP^y1^;gata1a:dsRed^sd2^)* (Yaniv et al., 2006) embryos revealed the presence of rostrocaudal blood flow in the SIA, whereas circulation in the SIV followed the directionality observed in the posterior cardinal vein (PCV; Fig. 1A, white arrows). Interestingly, we observed uniform dorsoventral flow (from the SIA to the SIV) in all of the ICVs (Fig. 1A, white arrows). In order to ascertain whether this functional characterization is supported by distinct expression of well-established arterial-venous markers, we imaged transgenic zebrafish embryos at 4-5 dpf. As seen in Fig. 1B, clear expression of the lymph-venous marker *lyve1* was detected in the PCV, thoracic duct (TD), SIV and ICVs, but not in the DA and SIA of *Tg(fli1:EGFP;lyve1:dsRed2^nz101^)* (Nicenboim et al., 2015) double transgenic embryos, indicating the venous identity of these vessels. By contrast, in the arterial specific *Tg(flt1_9a_cFos:GFP)^wz2^* reporter (Nicenboim et al., 2015) only the DA and SIA displayed strong GFP fluorescence (Fig. 1C), indicating that apart from the SIA, all components of the subintestinal plexus share a venous identity.

### Origin of the subintestinal vessels

**Fig. 2.**
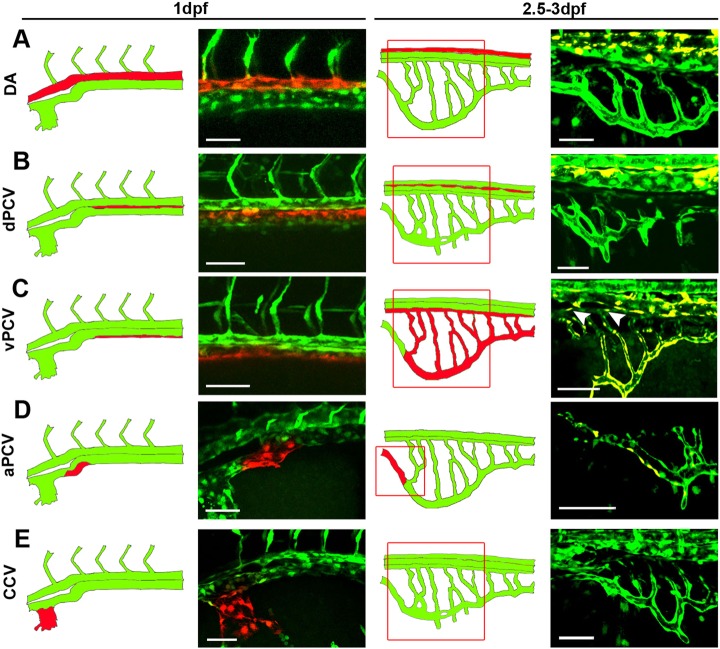
**PCV cells give rise to all components of the subintestinal plexus.** (A-E) Photoswitching of selected ECs was performed at 1 dpf in *Tg(fli1:gal4*;*uas:kaede)* embryos, and vessels were scored for the presence of red-labeled ECs at 2.5-3 dpf. Schematic illustrations of the corresponding confocal images are shown to the left. No photoconverted red cells were detected in the subintestinal plexus following photoswitching of the DA (A), dPCV (B) and CCV (E). Photoswitching of the vPCV (C) rendered red-labeled ECs in all components of the subintestinal plexus, including the SIA (arrowheads), whereas photoconverted ECs from the aPCV (D) were found exclusively in the rostral most part of the SIV. aPCV, anterior PCV; CCV, common cardinal vein; dPCV, dorsal PCV; vPCV, ventral PCV; for other abbreviations see legend to Fig. 1. Yellow channel denotes colocalization of green and red fluorescence. Scale bars: 50 µm. *n*_DA_=24, *n*_dPCV_=10, *n*_vPCV_=16, *n*_aPCV_=5, *n*_CCV_=11.

One of the major questions regarding the development of organ-specific vascular networks has to do with their embryonic origins. Given that the subintestinal plexus has been regarded as one of the possible origins of the GI tract vasculature, we decided to investigate the relative contribution of surrounding vessels to its formation, using photoconversion of ECs (Fig. 2; Fig. S1). We began by photoswitching all ECs in the DA of *Tg(fli1:gal4^ubs3^;uas:kaede^rk8^)* (Herwig et al., 2011) embryos at 1 dpf. Surprisingly, no red, photoconverted cells were detected in the subintestinal plexus at 2.5 dpf (Fig. 2A), suggesting that arterially derived ECs do not contribute to the formation of this vascular bed. We have recently uncovered the presence of specialized angioblasts within the ventral PCV (vPCV), which generate arterial, venous and lymphatic ECs (Nicenboim et al., 2015); therefore, we wondered whether these angioblasts serve as a source for late-forming vessels, such as the subintestinal plexus. Photoconversion of vPCV cells rendered red fluorescent cells in all vessels of the subintestinal plexus including the SIA (Fig. 2C, arrowheads), indicating the venous origin of all components of the digestive system vasculature. This was in contrast to cells of the dorsal PCV (dPCV); after photoswitching, these were found only in the intersegmental vessels of the trunk (Fig. 2B; data not shown). Interestingly, we noticed that the most rostral part of the SIV was not populated by red-labeled vPCV cells or by their progeny (Fig. 2C). To identify the origin of these cells, we photoconverted the left branch of the anterior portion of the PCV (aPCV), which also represents the SIV drainage point. As seen in Fig. 2D, red ECs were found in the rostral most part of the SIV in all analyzed embryos, indicating that the aPCV is not only connected to the SIV, but also contributes to its formation. Finally, no red cells were detected in the subintestinal plexus of 2.5 dpf embryos after photoconversion of the common cardinal vein (CCV) at 24 hpf (Fig. 2E), highlighting the vPCV angioblasts as the major source of ECs generating the subintestinal plexus.

### The subintestinal plexus gives rise to the vasculature of the digestive system

**Fig. 3.**
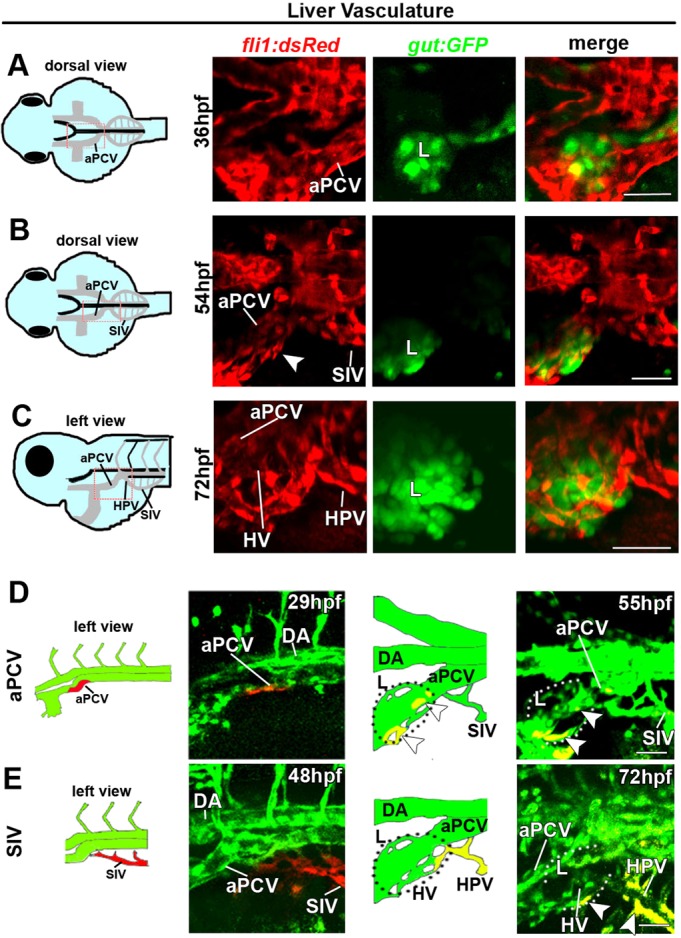
**The left subintestinal vein and aPCV give rise to the liver vasculature.** (A-C) Confocal images of *Tg(gut:GFP;fli1:dsRed)* double transgenic embryos at 36-72 hpf highlighting the vasculature (red) and the liver (L, green). (A) The aPCV on the left side of the embryo is found adjacent to the liver at 36 hpf. (B,C) ECs surround the liver by 54 hpf (B, arrowhead) and form the HVs by 72 hpf (C). The SIV drains into the liver via the HPV (C). (D,E) Photoswitching of ECs in the left branch of the aPCV (D) or the left SIV (E) was performed at 29 hpf in *Tg(fli1:gal4*;*uas:kaede)* embryos. (D) Red-labeled ECs from the left aPCV contribute to the liver vasculature (55 hpf, arrowheads). (E) Photoswitching of the left SIV at 48 hpf rendered red-labeled ECs in the HVs and the HPV (E, 72 hpf, arrowheads). aPCV, anterior PCV; HPV, hepatic portal vein; HV, hepatic vessels; L, liver; for other abbreviations see legend to Fig. 1. Yellow channel denotes colocalization of green and red fluorescence. Scale bars, 50 µm. *n*_aPCV_=4, *n*_SIV_=5.

In order to analyze the anatomical distribution of the subintestinal vessels with respect to the different organs of the digestive system, we first mated *Tg(fli1:dsRed)^um13^* (Covassin et al., 2009) and *Tg(gut:GFP)^s854^* (Field et al., 2003a) fish, in which GFP labels the endoderm and its derivatives. The spatiotemporal interaction of the plexus and the liver was examined by confocal microscopy between 36 hpf and 4 dpf (Fig. 3; Fig. S2). At 36 hpf, the left branch of the aPCV is detected in close proximity to the developing liver (Fig. 3A, ‘L’), which appears later on (54 hpf) wrapped by both the aPCV and the most rostral part of the SIV (Fig. 3B) (Isogai et al., 2001; Ober et al., 2003). At 72 hpf, the liver hepatic vessels (HVs) are fully established by ECs arising in the left aPCV and the anterior part of the left SIV, which drains into the liver as the hepatic portal vein (HPV; Fig. 3C). Towards 4 dpf, the hepatic vessels have acquired their typical reticular anatomy (Fig. S2A-A″). The contribution of the subintestinal plexus to the HVs was further addressed using *Tg(fli1:gal4;uas:kaede)* embryos. Photoconversion of the left branch of the aPCV at 29 hpf resulted in red cells populating the liver 24 h later (Fig. 3D, 55 hpf, white arrowheads). Likewise, photoconversion of the left SIV at 48 hpf rendered red cells that populated the HVs by 72 hpf (Fig. 3E, white arrowheads). Together, these results demonstrate that both the left aPCV and the rostral part of the SIV contribute to the liver vasculature and confirm that at these early stages of development, the hepatic vessels originate from the PCV or its derivatives, with no apparent arterial contribution.

**Fig. 4.**
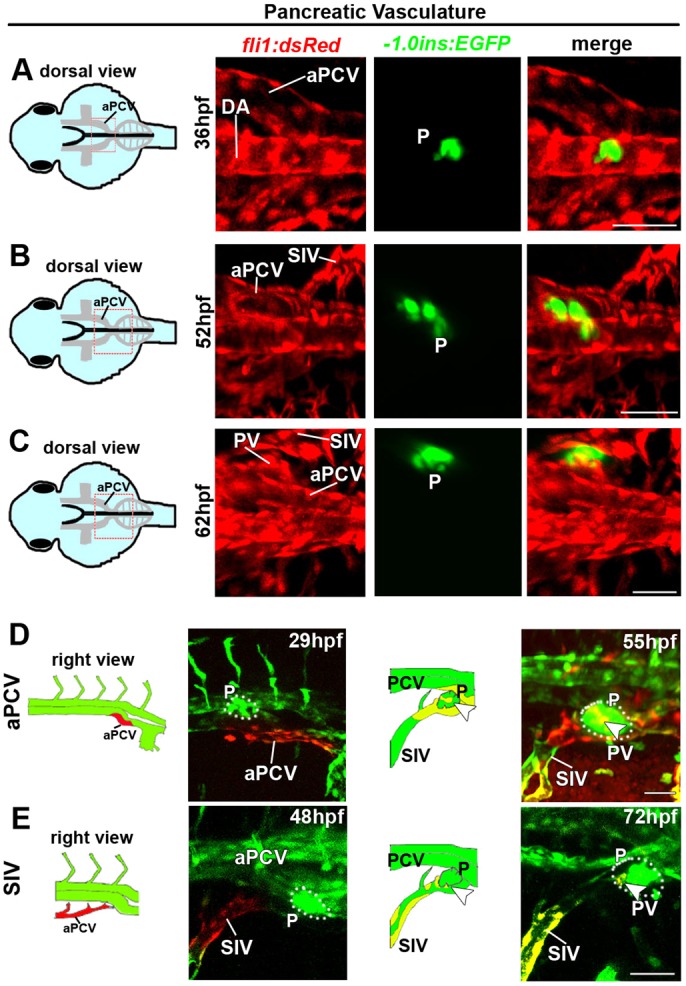
**The right subintestinal vein and aPCV give rise to the pancreatic vasculature.** (A-C) Confocal images of *Tg(-1.0ins:EGFP;fli1:dsRed)* embryos at 36-62 hpf highlighting the vasculature and the endocrine pancreas (P, green). (A) *insulin:EGFP^+^* cells are detected between the two branches of the aPCV at 36 hpf. (B,C) Gradual shift of *insulin:EGFP^+^* cells towards the right side of the midline. The most cranial ICV on the right side of the plexus (C) sends branches towards the nascent pancreas and forms pancreatic vessels (PV). (D,E) Photoswitching of ECs in the right branch of the aPCV at 29 hpf (D) or in the right SIV at 48 hpf (E) was performed in *Tg(fli1:gal4*;*uas:kaede;-1.0ins:EGFP)* embryos. (D) Red-labeled ECs from the right aPCV contribute to the pancreatic vessels (PV) (55 hpf, arrowhead). (E) Photoswitching of the right SIV at 48 hpf rendered red-labeled ECs in the pancreatic vessels (PV) (72 hpf, arrowheads). aPCV, anterior PCV; P, pancreas; PV, pancreatic vessels; for other abbreviations see legend to Fig. 1. Yellow channel denotes colocalization of green and red fluorescence. Scale bars, 50 µm. *n*_aPCV_=8, *n*_SIV_=11.

The vasculature of the intestinal bulb was examined in a similar manner (Fig. S3). At 36 hpf, the first ECs leave the floor of the PCV and sprout ventrally, right on top of the IB (Fig. S3A). Between 36 and 52 hpf, the intestine undergoes extensive morphogenesis and growth (Ng et al., 2005; Fig. S3A,B), until it acquires its final position on the left side at ∼72 hpf (Fig. S3C). Throughout this entire process, the IB is engulfed by the ICVs of the subintestinal plexus (Fig. S3B,C; Movie 1) and is entirely wrapped by the SIV, SIA and ICVs at later stages (Fig. S2B; Fig. S3C; Movie 1; Isogai et al., 2001). To study the pancreatic vasculature, *Tg(fli1:dsRed)* were mated with *Tg(-1.0ins:EGFP)^sc1^* (diIorio et al., 2002) fish, in which cells of the endocrine pancreas are highlighted in green (Fig. 4; Fig. S2C). Confocal imaging of double transgenic embryos confirmed the gradual shift of the pancreatic anlage from a medial position at 36 hpf towards the right side of the midline (Field et al., 2003b), beneath the right aPCV (Fig. 4A-C). By 62 hpf, the pancreatic anlage is positioned between the right SIV and the right aPCV; the most cranial ICV on the right side of the plexus interconnects the SIV and the SIA to form the pancreatic vessels (Fig. 4C; Fig. S2C). Photoconversion of either the right aPCV at 29 hpf or the most rostral part of the SIV at 48 hpf resulted in red labeling of the pancreatic vessels at 55 and 72 hpf, respectively (Fig. 4D,E, arrowheads). In addition, the direct contribution of ECs from the aPCV and SIV to the pancreatic vasculature was tracked *in vivo.* After focused photoswitching on *Tg(fli1:gal4*;*uas:kaede;-1.0ins:EGFP)* embryos, time-lapse confocal microscopy was used in order to follow the generation of the pancreatic vessels from the aPCV and SIV between 54 and 73.5 hpf (Movie 2). Taken together, these data indicate that the vasculature of at least three different organs of the digestive system originates in the PCV, either directly or through the intermediate subintestinal plexus. Furthermore, these findings highlight the plasticity of the vPCV angioblasts and their ability to contribute to different vessel types.

### Different mechanisms of EC migration underlie the formation of the subintestinal plexus

**Fig. 5.**
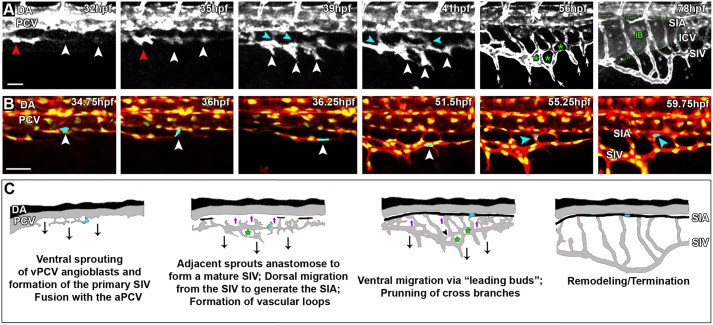
**Formation of the subintestinal plexus involves different mechanisms of EC migration.** (A) Snapshots from a time-lapse sequence of a *Tg(fli1:dsRed)* embryo, showing individual sprouts arising from the vPCV (32 hpf, white arrowheads), which quickly anastomose and fuse with a caudal projection of the aPCV (red arrowhead) to generate the primary SIV. Sprouts arising along the primary SIV elongate ventrally and fuse to generate the mature SIV (35-41 hpf, white arrowheads). Concomitantly, ECs from the SIV migrate dorsally to generate the SIA (39-41 hpf, light-blue arrowheads). The mature SIV migrates ventrally through collective migration guided by leading buds (56 hpf, white arrows), while fusion of angiogenic sprouts originating in the primary SIV generates vascular loops (56 hpf, green asterisks). Retraction of the leading buds, along with pruning of the cross-branches forming the vascular loops, render the stereotypical basket shape (78 hpf) that engulfs the intestinal bulb (IB). (B) Snapshots from a time-lapse sequence of a *Tg(fli1:dsRed;fli1:nGFP)* embryo showing the migration route taken by vPCV angioblasts. A single angioblast (light blue) initially residing in the vPCV (34.75 hpf, white arrowhead) sprouts ventrally (36 hpf, white arrowhead) and incorporates into the primary SIV (36.25-51.5 hpf, white arrowhead). Later on, the same cell migrates dorsally (55.25 hpf, light blue arrowhead), eventually becoming incorporated into the SIA (59.75 hpf, light blue arrowhead). (C) Schematic illustration depicting the different steps involved in formation of the subintestinal plexus: ventral sprouting and ventral migration (black arrows); cells ‘lagging back’ followed by dorsal migration (purple arrows); vascular loops (green asterisks); and vessel pruning (black arrowhead). Scale bars: 50 µm.

In order to investigate the dynamics of formation of the subintestinal plexus, we live-imaged *Tg(fli1:dsRed;fli1:nGFP^y7^)* double transgenic embryos in time lapse, between 30 and 90 hpf (Fig. 5A,C; Movie 1). At 32 hpf, we detected short ventral sprouts arising from the vPCV, which quickly anastomose to generate a single vessel (Fig. 5A, 32 and 35 hpf, white arrowheads) that extends rostrally and fuses with a caudal projection arising from the aPCV (Fig. 5A, 32 and 35 hpf, red arrowhead). This newly formed primary SIV undergoes extensive remodelling that involves the fusion of angiogenic sprouts (Fig. 5A, 35-41 hpf, white arrowheads) and formation of vascular loops (Fig. 5A, green asterisks), until the establishment of a mature SIV is achieved. The ventral migration of the mature SIV is then guided by three to five short ‘leading buds’ (Fig. 5A, 56 hpf, arrows), which later on retract, rendering the stereotypical subintestinal basket shape by ∼70-80 hpf (Fig. 5A,C; Movie 1). During these last steps, active pruning of the cross-branches is also observed (Lenard et al., 2015). Along with the ventral expansion of the plexus, we detected ECs migrating dorsally from the SIV to populate the SIA (Fig. 5A, 39-41 hpf, light-blue arrowheads; Fig. 5C). In order to ascertain whether the same vPCV cells that populate the SIV also become incorporated into the SIA, we tracked the migration of individual ECs in *Tg(fli1:dsRed;fli1:nGFP)* embryos between 30 and 60 hpf (Fig. 5B,C; Movie 3). We found that vPCV angioblasts (Fig. 5B, 34.75 hpf; Fig. 5C) leave the PCV at ∼36 hpf and migrate ventrally to become incorporated into the primary SIV (Fig. 5B, 36-51.5 hpf; Fig. 5C). Some of these cells will then engage in dorsal migration to end up populating the SIA (Fig. 5B, 55.25-59.75 hpf, light blue arrowhead; Fig. 5C). Altogether, our time-lapse analyses reveal that vPCV angioblasts contribute to all components of the subintestinal plexus through two different phases of migration. Initially, they sprout from the PCV, anastomose and migrate ventrally to generate a mature SIV. While the ventral expansion of the SIV and ICVs takes place, some cells lag back slightly and migrate dorsally to generate the arterial component of the plexus (Fig. 5C, purple arrows; Movie 3).

**Fig. 6.**
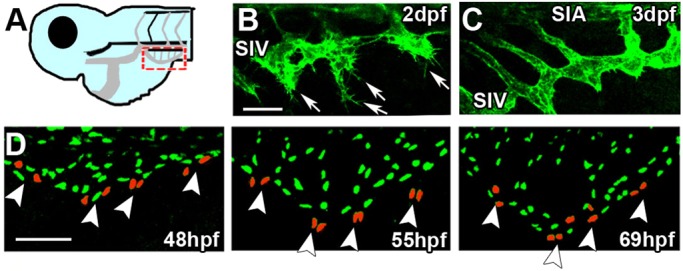
**Establishment of the SIV involves leading bud-guided collective migration of ECs.** (A) Dashed red box in the diagram shows approximate location of regions imaged in B-D. (B) Actin-rich filopodia (arrows) are detected at the SIV migration front in *Tg(Lifeact:GFP)* embryos at 2 dpf. (C) As the plexus reaches its stereotypical basket shape at 3 dpf, all filopodia retract. (D) Distribution of EC nuclei in *Tg(fli1:nGFP)* embryos demonstrates that leading buds consist of paired ECs (48-55 hpf, arrowheads), rather than of a single tip cell. Towards the end of the process, leading buds retract and are incorporated into the SIV (69 hpf, arrowheads). Scale bars: 50 µm.

During active angiogenesis, formation and growth of new sprouts involve coordination between opposing tip and stalk cell behaviors, a process that is tightly regulated by the VEGF and Notch signaling pathways (Hellström et al., 2007; Siekmann and Lawson, 2007; Jakobsson et al., 2010). Interestingly, however, our live-imaging analyses revealed a step of collective migration involved in formation of the subintestinal plexus, whereby the SIV extends ventrally as a single unit, and not through the classic tip/stalk cell mechanism. By following actin dynamics in ECs using *Tg(fli1:Lifeact-GFP)^wz4^* embryos (Fig. 6A-C), we detected the presence of numerous actin-rich filopodia in the leading buds at the migration front (Helker et al., 2013; Fig. 6B, arrows), a behavior similar to that observed in ISV tip cells (Phng et al., 2013) and in the vascular front of the postnatal mouse retina (Gerhardt et al., 2003). These features were apparent throughout the entire phase of formation of the mature SIV and its subsequent ventral expansion, but were no longer evident as ventral migration ceased by 3 dpf (Fig. 6C). Examination of the spatial distribution of the EC nuclei in the SIV of *Tg(fli1:nGFP)* embryos (Fig. 6D; Movie 4) revealed that ∼80% of the leading buds consisted of paired ECs positioned in parallel to each other, rather than of a single tip cell (Fig. 6D, 48-55 hpf, arrowheads). Moreover, none of these nuclei exhibited dominancy over its neighbor, and both remained adjacent to each other throughout the entire phase of ventral migration. As ventral expansion of the plexus approached termination, ECs constituting each of the leading buds incorporated into the SIV or migrated dorsally to populate the ICVs (Fig. 6D, 69 hpf, arrowheads; Movie 4).

**Fig. 7.**
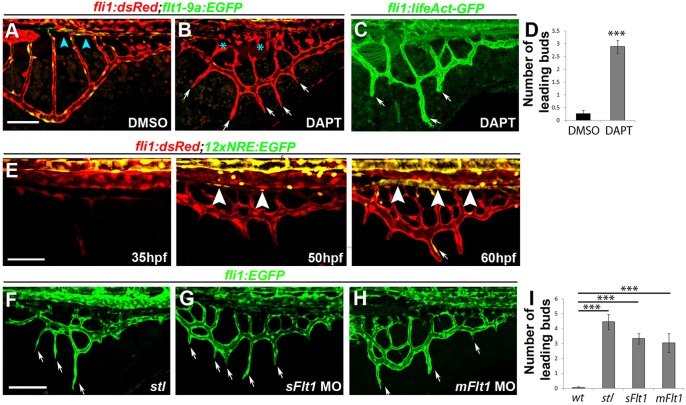
**Notch activity is required for final remodeling of the subintestinal plexus but is dispensable for its initial development.** (A-D) The subintestinal plexus of 72 hpf *Tg(fli1:dsRed;flt1_9a_cFos:GFP)* embryos treated with DAPT between 24 and 72 hpf shows the presence of ectopic leading buds (B,C, arrows; D), malformed SIA (B, asterisks) and active filopodia along the SIV (C, arrows). *n*_DMSO_=29; *n*_DAPT_=36. ***P<0.001. (E) Spatiotemporal characterization of Notch signaling activation during development of the subintestinal plexus, as detected in *Tg(fli1:dsRed;12xNRE:EGFP)* double transgenic embryos. EGFP is detected in the SIA starting at 50 hpf (arrowheads) and in the leading buds of 60 hpf embryos (arrow). (F-I) Downregulation of *flt1* results in ectopic leading buds along the SIV of *stl* mutants (F, arrows), *sFlt1* (G, arrows) and *mFlt1* (H, arrows) morphants, quantified in I. Yellow channel denotes colocalization of green and red fluorescence. Scale bars: 50 µm. *n*_WT_=16, *n_stl_*=13, *n_mFlt_*=27, *n_sFlt_*=18. ***P<0.001. Error bars represent s.e.m.

Given the importance of Notch signaling in coordinating vessel sprouting in other contexts, we assessed the role of this pathway during formation of the subintestinal plexus. We began by analyzing the effects of the γ-Secretase inhibitor DAPT following treatment of *Tg(fli1:dsRed;flt1_9a_cFos:GFP)* embryos between 24 and 72 hpf (Fig. 7). Unlike DMSO-treated control siblings, in which the stereotypical basket shape has been consolidated by 3 dpf (Fig. 7A), in embryos raised in the presence of 100 µM DAPT the retraction of the leading buds during the final stages of SIV remodeling was inhibited (Fig. 7A-D). In addition, we detected defects in formation of the SIA, including marked reduction in the expression of the arterial marker *flt1_9a_cFos:GFP* (Fig. 7B, asterisks), suggesting that Notch signaling is required for proper formation of a functional SIA, including its arterial differentiation. In addition to the impaired retraction of leading buds induced by DAPT treatment, we also detected persistent filopodia along the entire SIV by 72 hpf (Fig. 7C, arrows). These findings are in line with previous reports describing excessive filopodia formation following DAPT treatment in the ISVs of the zebrafish trunk (Leslie et al., 2007). To corroborate these results further, we examined the pattern of Notch activation in the subintestinal plexus using the *Tg(EPV.Tp1-Mmu.Hbb:EGFP)^ia12^* reporter line (12xNRE:EGFP) (Fig. S4). This reporter, which consists of 12 repeats of Notch-responsive elements driving EGFP expression (Moro et al., 2013), is specifically responsive to Notch signaling, as manifested by its dose-dependent downregulation in response to DAPT treatment (Fig. S4). Analysis of transgenic embryos between 35 and 60 hpf revealed Notch activation in the DA and arterial ISVs (Fig. 7E; data not shown; Quillien et al., 2014). Nonetheless, no Notch-derived fluorescence was observed in the subintestinal plexus until ∼50-60 hpf, when Notch-positive cells were detected in the SIA (Fig. 7E, 50 and 60 hpf, arrowheads), further supporting the arterial identity of this vessel (Lawson et al., 2001; Lawson and Weinstein, 2002; Swift and Weinstein, 2009), as opposed to the rest of the components of the subintestinal basket (Fig. 1). In addition to the SIA, clear Notch-derived EGFP fluorescence was detected in a few cells within the retracting leading buds during the final stages of remodeling of the plexus (Fig. 7E, 60 hpf, arrow). These results are in line with the phenotypes resulting from DAPT treatment and suggest that the role of Notch signaling is restricted to the late steps of resolution of the subintestinal angiogenic process, whereas it is dispensable for the earlier development of the plexus.

Interestingly, the ectopic SIV sprouts observed after DAPT treatment were reminiscent of those seen in *stalactite* (*stl*) mutants (Avraham-Davidi et al., 2012; Fig. 7F, arrows). In these embryos, ectopic angiogenesis results from the absence of apoprotein B (apoB)-containing lipoproteins, which in turn induces a significant reduction in the levels of the decoy receptor Vegfr1 (Flt1; Avraham-Davidi et al., 2012). To analyze the putative involvement of Flt1 during formation of the subintestinal plexus, we downregulated both the membrane and the soluble isoforms of Flt1 using antisense morpholino oligonucleotides (MOs) (Zygmunt et al., 2011). Although in WT siblings all leading buds have retracted by 3 dpf and a ‘clean’ basket-shaped plexus is observed (Fig. 7A), these buds fail to remodel and retract following downregulation of Flt1 in *stl* mutants, *mFlt1* or *sFlt1* morphants (Fig. 7F-H, arrows; Fig. 7I). Interestingly, *lyve1:dsRed* fluorescence was detected in the ectopic leading buds of both *stl* mutants and *flt1* morphants (Fig. S5), indicating that the venous identity of these sprouts is not dependent on Flt1. Altogether, these results suggest that Notch and Flt1 participate in the remodeling of the subintestinal plexus, which involves retraction of venous leading buds, but are not required for its initial development.

### Molecular cues controlling the formation of the subintestinal vessels

**Fig. 8.**
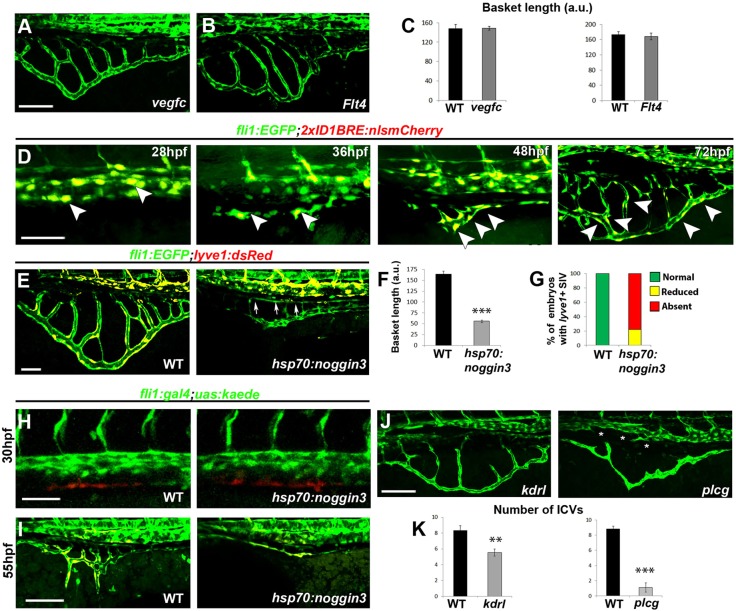
**Molecular mechanisms controlling formation of the subintestinal plexus*.*** (A-C) Subintestinal vessels form normally in *vegfc* (A,C) and *flt4* (B,C) mutants. *n*_vegfc_=19; *n*_Flt4_=5. (D) Activation of BMP signaling is detected in the PCV (28 hpf, arrowheads) and SIV (36-72 hpf, arrowheads) of *Tg(2xID1BRE:nlsmCherry)* embryos. (E-G) Heat shock induction of *noggin3* expression in *Tg(fli1:EGFP; lyve1:dsRed; hsp70l:noggin3)* embryos inhibits ventral migration of the plexus, as manifested by reduced basket length when compared with *Tg(fli1:EGFP;lyve1:dsRed)* (WT) embryos (E,F). The SIA forms normally (E, arrows). Cells in the SIV of *noggin3*-overexpressing embryos fail to upregulate the lymph-venous marker *lyve1* (E,G). (H,I) The vPCV of *Tg(fli1:gal4;uas:kaede)* (WT) and *Tg(fli1:gal4;uas:kaede;hsp70l:noggin3)* double transgenic embryos was photoconverted at 30 hpf (H). At 55 hpf, red/yellow-labeled ECs were detected in the SIV of both WT and *hsp70l:noggin3*-overexpressing embryos (I). (J,K) Impaired Vegf signaling results in reduced number of ICVs in *kdrl* and *plcg* mutants (J,K). The SIA is absent in *plcg* mutants (J, asterisks).Yellow channel denotes colocalization of green and red fluorescence. Scale bars: 50 µm. *n_vegfc_*=19; *n_Flt4_*=5; *n_noggin3_*=11; *n_kdrl_*=13; *n_plcg_*=9. **P<0.01; ***P<0.001. Error bars represent s.e.m.

The intricate paths involved in shaping the subintestinal vasculature prompted us to enquire into the molecular mechanisms underlying the different phases of this process. Given that venous sprouting was shown to require Vegfc signaling (Covassin et al., 2006; Küchler et al., 2006; Hogan et al., 2009a), we examined the involvement of Vegfc and Vegfr3/Flt4 in sprouting of ECs from the vPCV and formation of the subintestinal plexus. Analysis of *vegfc^um18^* (Villefranc et al., 2013) and *flt4^um203^* (Kok et al., 2015) mutants revealed no major defects in venous sprouting from the vPCV or in the shape or length of the subintestinal basket (Fig. 8A-C), suggesting that the Vegfc-Flt4 axis does not play a major role in this process. Recently, the Bone Morphogenetic Protein (BMP) signaling pathway has been implicated in sprouting from the axial vein (Wiley et al., 2011). Accordingly, we examined the role of BMP in shaping the subintestinal vasculature. We first analyzed BMP activity between 28 and 72 hpf in *Tg(2xID1BRE:nls-mCherry)ia17*, in which a BMP-responsive element (BRE) containing multiple Smad-binding sites induces expression of nuclear mCherry, in response to Smad1/5/8 activation (Moro et al., 2013). Prior to ventral sprouting from the PCV, BMP-derived fluorescence was detected in EC nuclei within the axial vessels (Fig. 8D, 28 hpf, arrowheads). Later on, clear labeling was seen in the primary SIV (Fig. 8D, 36 hpf, arrowheads) and throughout the SIV and ICVs (Fig. 8D, 48 and 72 hpf, arrowheads). Nevertheless, no BMP activation was observed in the SIA. These results indicate the involvement of BMP signaling in ventral migration of the vPCV angioblasts and in maintenance of venous fate of the SIV. In order to ascertain whether BMP is not only expressed in ECs forming the SIV, but also plays a role in this process, we examined the formation of the plexus in *Tg(fli1:EGFP;lyve1:dsRed;hsp70l:noggin3)* embryos, generated by mating *Tg(fli1:EGFP;lyve1:dsRed)* fish with the *Tg(hsp70l:noggin3)^fr13^* (Chocron et al., 2007) line, in which forced expression of the BMP antagonist *noggin3* is induced by heat shock. Although venous sprouting from the vPCV appears to occur normally in these embryos, as evident from the fact that a primary SIV is established at the relevant developmental stages, overexpression of *noggin3* completely inhibited the ventral migration of the subintestinal plexus, resulting in a significantly dorsalized basket (Fig. 8E,F). Interestingly, heat shock induction of *noggin3* in *Tg(fli1:EGFP;lyve1:dsRed;hsp70l:noggin3)* embryos also resulted in loss of *lyve1*-derived dsRed fluorescence in the subintestinal basket of 82% of the treated embryos (Fig. 8E,G). This effect might be attributed to a potential role for the BMP signaling pathway in induction and/or maintenance of venous fate in the vPCV angioblasts. Alternatively, reduction of *lyve1*-derived fluorescence in the SIV might reflect a different origin of the ECs forming these vessels, under *noggin3* overexpression. To distinguish between these two possibilities, we crossed *Tg(hsp70l:noggin3)* with *Tg(fli1:gal4*;*uas:kaede)* fish, heat shocked their progeny at 26 hpf, and photoconverted the vPCV angioblasts at 30 hpf (Fig. 8H). At 55 hpf, the SIVs of both control and *noggin3*-overexpressing embryos displayed red/yellow fluorescence (Fig. 8I), suggesting that although the vPCV angioblasts are still able to leave the PCV and generate a primary SIV, they neither upregulate the expression of venous markers nor engage in collective ventral migration, highlighting the BMP signaling pathway as a major player during the initial phases of formation of the subintestinal plexus.

### ‘Arterial’ signals control the development of the SIA

Although ventral migration of the SIV was regulated by BMP, perturbations in this pathway had no apparent effect on formation of the SIA (Fig. 8E, arrows). We therefore decided to explore the involvement of well-established ‘arterial’ circuits in formation of this vessel. We began by imaging *kdrl^y17^* mutants, which carry a mutation in one of the zebrafish Vegf receptor-2 orthologs, previously shown to result in arterial-specific defects (Covassin et al., 2006). Assessment of the subintestinal plexus in these embryos revealed that both the sprouting of angioblasts from the vPCV and the subsequent formation of the SIV take place normally (Movie 5). Nonetheless, the subintestinal plexus of these embryos was characterized by a reduced number of ICVs compared with their phenotypically wild-type (WT) siblings (Fig. 8J,K; Movie 5). These findings suggest a role for VEGF signaling in the ‘arterial’ phase of the process, namely the dorsal migration of ECs from the SIV to generate the SIA. To confirm this notion, we analyzed the formation of the SIA in *plcg1^y10^* mutants (Lawson et al., 2003), which lack Phospholipase C gamma-1 (*plcg*), a downstream effector of VEGF/Vegfr2 signaling (Takahashi et al., 2001). These mutants display clear defects in arterial development but exhibit normal venous and lymphatic sprouting (Lawson et al., 2003; Lim et al., 2011; Nicenboim et al., 2015). The initial formation and the ventral migration of the SIV were normal in *plcg1* mutants (Movie 6), but we detected a marked decrease in the number of ICVs and a complete absence of the SIA in all examined embryos (Fig. 8J,K; Movie 6), indicating a key role for the *kdrl-plcg* axis in the dorsal migration phase of the subintestinal plexus formation.

## DISCUSSION

**Fig. 9.**
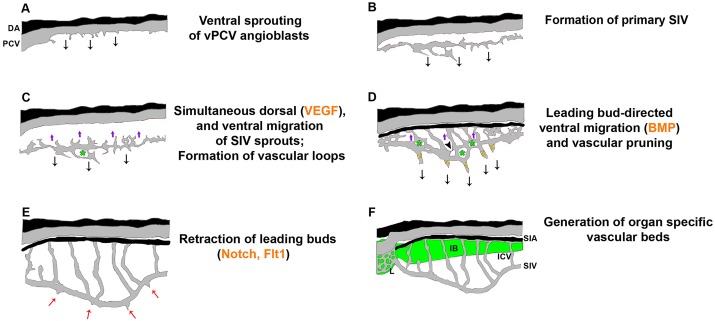
**Molecular and cellular mechanisms underlying the formation of the GI vasculature.** (A-F) Schematic illustrations depicting the different steps and molecular cues involved in development of the subintestinal plexus. Black arrows, ventral sprouting and ventral migration; purple arrows, ECs ‘lagging back’ and dorsal migration; black arrowhead, vessel pruning; green asterisks, vascular loops; red arrows, retraction of leading buds; green shading, GI tract.

We provide here a comprehensive study of the different steps underlying the formation of the zebrafish subintestinal plexus and its relationship to the vascularization of the GI tract (Fig. 9). Our data track the embryonic origins and the derivatives of each component of the subintestinal vessel network and characterize the molecular circuits governing morphological changes that model the plexus into its final stereotypical shape. Serving as the primary source of the gastrointestinal vasculature, our results place the subintestinal plexus as an ideal model to study the interactions between organs and their specific vascular beds.

Using live imaging of transgenic zebrafish, we show that formation of the subintestinal plexus takes place through ventral sprouting of specialized angioblasts from the floor of the PCV, which quickly anastomose and generate a primary SIV. EC differentiation and collective ventral migration of the SIV are accompanied by incorporation of cells into the SIA, a phase that involves dorsal migration of single cells from the SIV. These findings highlight the plasticity of the vPCV angioblasts and demonstrate their ability not only to generate arterial, venous and lymphatic ECs (Nicenboim et al., 2015), but also to contribute to mature vessels of at least three different organs (intestine, liver and pancreas), displaying significantly different features (Nolan et al., 2013).

Our previous results demonstrated that upregulation of Prox1a in the vPCV angioblasts or in their progeny determines their specification towards a lymphatic fate (Nicenboim et al., 2015). By contrast, we never detected Prox1a^+^ cells migrating ventrally to populate the subintestinal vessels (data not shown), highlighting the specific contribution of *prox1a^+^* cells to the lymphatic endothelium and not to other vascular beds. Based on these results, we postulate that the vPCV angioblasts bear the potential to generate multiple cell fates within the endothelial cascade. Nonetheless, *in vivo* they generate progeny based upon the signals to which they are exposed and the specific developmental stage. Consequently, vPCV cells located in close proximity to the endoderm are more likely to acquire a lymphatic fate following induction by the endoderm-secreted Wnt5b (Nicenboim et al., 2015) than vPCV cells that are located at more rostral positions, where the expression of Wnt5b is not detected. This hypothesis is further supported by the fact that as development proceeds there is a shift in the fate acquired by vPCV cells from a population of parachordal cells (PACs) towards the subintestinal plexus (Nicenboim et al., 2015). Future experiments will be required to ascertain whether the vPCV angioblasts retain their plasticity as development proceeds, enabling the generation of different derivatives following exposure to diverse inducing signals.

Our genetic analyses show that ECs forming the subintestinal plexus respond to specific cues, which differentially guide their migration during each phase of the process. Through the use of genetic manipulations and transgenic reporters, we demonstrate a clear role for the BMP signaling pathway in ventral migration and expansion of the plexus. Our data are in line with the findings reported by Wiley et al. (2011) showing that BMP signaling is necessary for the formation of the venous-derived caudal vein plexus (CVP). In that study, ECs in the CVP were shown to express the BMP receptors *bmpr2a* and *bmpr2b* and to respond to BMP signaling. Furthermore, Bmp2b overexpression at 2.5 dpf resulted in ectopic sprouts in the SIV, suggesting that this vessel is responsive to BMP signaling (Wiley et al., 2011). In accordance with these results, we detected clear BMP activation in ECs of the SIV (Fig. 8D) during the ventral migration and expansion of the basket. By contrast, while antagonizing BMP signaling inhibited the ability of sprouts from the axial vein to make connections and to form a proper CVP, BMP signaling was neither required for the initiation of vPCV ventral sprouting in the yolk area nor for the establishment of a primitive SIV, thereby suggesting that this step is controlled by additional, yet unknown, molecular signals. Interestingly, forced overexpression of *noggin3* rendered a primary SIV that was not labeled by the lymph-venous marker *lyve1*, indicating an additional requirement for the BMP signaling pathway for proper specification and/or maintenance of venous fate in the vPCV angioblasts. We have recently identified the endoderm-secreted Wnt5b as a novel inductive signal promoting the ‘angioblast-to-lymphatic’ specification in the vPCV angioblasts (Nicenboim et al., 2015). In turn, Bmp2b has been shown to regulate lymphatic cell fate specification negatively (Dunworth et al., 2014), suggesting that the interplay between these two signaling pathways is instrumental for establishing the balance between venous versus lymphatic ECs. The exact molecular mechanism underlying this crosstalk remains to be clarified.

Although ventral migration of the SIV was BMP dependent, dorsal migration of ECs from the SIV to generate the SIA was controlled by VEGF signaling. In both *kdrl* and *plcg1* mutants, the number of ICVs was reduced, and severe defects were observed in the formation of the SIA. Interestingly, the dorsal migration of ECs from the SIV to generate the SIA resembles morphologically the sprouting of arterial ISVs in the developing trunk (Isogai et al., 2003). Moreover, a common inhibitory effect over dorsal sprouting is seen in *kdrl* and *plcg1* mutants, in both the trunk ISVs (Lawson et al., 2003) and the ICVs of the subintestinal plexus (Fig. 8). This similarity is intriguing, considering that inhibition of Notch signaling affected the trunk and the subintestinal vasculature in different ways; although DAPT treatment induced an increased number of ISV tip cells, dorsal sprouting from the SIV and formation of the ICVs was normal. Yet, reduced Notch signaling affected mainly the final steps of remodeling of the subintestinal plexus, primarily inhibiting the retraction of the venous leading buds. This is in agreement with previous data showing that Notch activation via overexpression of Dll4 results in a reduction in the number of venous sprouts during secondary sprouting from the PCV (Hogan et al., 2009b). Likewise, Dll4-containing nucleosomes were shown to prompt human microvascular endothelial cells to lose their filopodia and retract (Sharghi-Namini et al., 2014), supporting a differential role for Notch signaling during venous versus arterial sprouting processes.

Given the molecular complexity of the circuits regulating the proper formation of the subintestinal plexus, it seems likely that additional factors, such as blood circulation, might play a role in this process. Although the role of blood flow during formation of the subintestinal plexus has remained controversial (Cermenati et al., 2008; Montero-Balaguer et al., 2009; Lenard et al., 2015), our preliminary data suggest that heart-beat arrest affects sprouting from the PCV and initial formation of the SIV (data not shown). Interestingly, in addition to the well-established role of the Notch signaling pathway in EC differentiation, specification, sprouting and migration (Phng and Gerhardt, 2009), it has also been shown to mediate the angiogenic effects of blood flow, including vessel identity and remodeling (Jones et al., 2006). Therefore, the subintestinal phenotypes resulting from the absence of blood flow might potentially be mediated by Notch signaling. Alternatively, they could result from indirect, secondary effects caused by heart failure, and later on by hypoxia. Given that the subintestinal plexus forms relatively late, as opposed to the primary intersegmental vessels that are only minimally affected by the lack of circulation, we cannot exclude the possibility that the second hypothesis is correct. Further *in vivo* experiments enabling segregation between direct versus indirect effects of absent blood flow will be required in order to answer this question.

Our live-imaging analyses revealed that ventral expansion of the plexus involves a phase of collective migration of the SIV via leading buds, which retract as the basket reaches its final shape. Interestingly, inhibition of both Notch and Flt1 activity resulted in the inability of the leading buds to retract. Impaired Notch signaling rendered embryos displaying ectopic SIV sprouts, as described following downregulation of the Notch ligand Delta-like 1 (Rodriguez et al., 2012). Moreover, our findings are in line with previous reports indicating the inhibitory effect of the Notch-Dll4 pathway on remodeling and regression of blood capillaries in the mouse retina model of oxygen-induced retinopathy (Lobov et al., 2011). A role for the Notch pathway in remodeling of the subintestinal plexus is further supported by the spatiotemporal pattern of Notch activity that was restricted to the SIA and to cells in leading buds, at a stage corresponding to remodeling of the plexus. In addition to Notch signaling, downregulation of both *mFlt1* and *sFlt1* also resulted in the presence of ectopic leading buds along the SIV, in agreement with the phenotype of *stl* mutants (Avraham-Davidi et al., 2012), suggesting a specific role for Flt1 in remodeling of the plexus through retraction of the leading buds. Altogether, our results highlight certain similarities between the mechanisms underlying formation of the subintestinal plexus and the ‘classic’ process of tip/stalk cell coordination: (1) the angiogenic front is highly active, with extensive filopodial formation; (2) this process ceases once the vessels form and acquire their final shape; and (3) there appears to be a feedback interaction between Notch and Vegf; loss of Notch and loss of Flt1 (similar to gain of Vegf) result in similar phenotypes. The main difference in the formation of these two vascular beds resides in the particular cell behaviors; SIV formation involves a collective movement whereby a single vessel migrates as one unit, versus the traditional sprouting mechanism that takes place during development of the trunk ISVs.

Interestingly, we found that the leading buds, which lead the collective ventral migration of the SIV, consisted mostly of two tip cells. These cells were found to overlap during most of the process of ventral expansion of the mature SIV. Similar results were reported for different sprouting assays, including human umbilical endothelial cells, mouse retina and mouse embryonic back vessels (Pelton et al., 2014). In all these cases, the majority of the angiogenic sprouts were shown to consist of two overlapping, parallel cells, a phenomenon that could not be accounted for by a transient overlap resulting from a switch in position between the cells. In the future, it will be interesting to investigate whether this mechanism of vessel sprouting is specific for venous ECs. Our *in vivo* data support this idea and place the subintestinal plexus as an advantageous model for the study of this and other cellular and molecular mechanisms controlling the interaction between tip/stalk and tip/tip cells during venous sprouting.

As a whole, the results presented here establish the subintestinal plexus as a model for the study of organ-specific vessel development. This process, which includes the specification of angioblasts towards venous and arterial fates, along with differential mechanisms of EC migration and subsequent specification within a tissue-specific niche, is highly relevant to our understanding of blood vessel formation and wiring during disease states and tissue regeneration.

## MATERIALS AND METHODS

### Zebrafish husbandry and transgenic lines

Zebrafish were raised by standard methods (Avraham-Davidi et al., 2012) and handled according to the guidelines of the Weizmann Institute Animal Care and Use Committee. The *Tg(fli1:EGFP)^yl^*, *Tg(fli1:nGFP^y7^)* (Yaniv et al., 2006), *Tg(fli1:dsRed)^um1^* (Covassin et al., 2009), *Tg(lyve1:dsRed2)^nz101^* (Okuda et al., 2012), *Tg(fli1:gal4^ubs3^*;*uasKaede^rk8^)* (Herwig et al., 2011), *Tg(gata1a:dsRed)^sd2^* (Yaniv et al., 2006), *vegfc^um18^* (Villefranc et al., 2013), *flt4^um203^* (Kok et al., 2015), *Tg(hsp70l:noggin3)^fr13^* (Chocron et al., 2007), *Tg(flt1_9a_cFos:GFP)^wz2^* (Nicenboim et al., 2015), *Tg(gut:GFP)^s854^* (Field et al., 2003a), *Tg(-1.0ins:EGFP)^sc1^* (diIorio et al., 2002), *kdrl^y17^* (Covassin et al., 2006), *Tg(2xID1BRE:nlsmCherry)ia17* (Moro et al., 2013), *stl* (Avraham-Davidi et al., 2012) and *plcg1^y10^* (Lawson et al., 2003) were described elsewhere. Genotypes of *vegfc^um18^* and *flt4^um203^* mutants were verified as described before (Villefranc et al., 2013; Kok et al., 2015). The *Tg(fli1:LifeAct-GFP)^w4^* was generated by cloning the LifeAct sequence (Riedl et al., 2008) into the TolFliepDest vector using the Gateway methodology (Villefranc et al., 2007). The Notch-responsive GFP reporter line, *Tg(EPV.Tp1-Mmu.Hbb:EGFP)^ia12^* (12xNRE:EGFP) was generated by injection of a construct composed of six copies of the Epstein-Barr Virus Tp1 enhancer, each containing two Rbp-Jk binding sites, for a total of 12 Notch-responsive elements, placed in front of a murine beta-globin basal promoter driving EGFP (Parsons et al., 2009).

### Manipulation of zebrafish embryos

#### Morpholino injection

The *sFlt1* 5′-GCCGCTATAAAGAATAAGGGCCTGA-3′ (5 ng) and *mFlt1* 5′-CAGCAGTTCACTCACATCTCCGTTC-3′ (5 ng) (Zygmunt et al., 2011) antisense morpholino oligonucleotides (Gene-tools) were resuspended and injected as described by Ben Shoham et al. (2012).

#### Heat shock

*Tg(hsp70l:noggin3)* embryos at 26 hpf were heat shocked at 37°C for 40 min and scored for vascular phenotypes at 2 or 3 dpf. Genotyping was carried out using the *hsp70l* forward primer (5′-CATGTGGACTGCCTATGTTCATC-3′) and the *noggin3* reverse primer (5′-GGTGGCCAGGAAATACGGGATG-3′).

#### DAPT experiments

Egg water containing DAPT was prepared by diluting fresh InSolution γ-Secretase Inhibitor IX (Merck Millipore) in fish water. Embryos were treated with 10 or 100 µM DAPT or DMSO (1:250) for the indicated time period and examined at 48 or 72 hpf.

Photoswitching of *Tg(fli1:gal4;uasKaede)* embryos was performed using a 405 nm laser as described (Nicenboim et al., 2015). Embryos were analyzed at 24-48 h after photoswitching. The percentage of embryos with red fluorescent cells in each vessel of the subintestinal plexus was calculated. Embryos with no fluorescence or with gross vascular morphological defects were excluded from quantification.

Quantification of phenotypes, including number of ICVs, number of ectopic SIV sprouts and length of the subintestinal basket (the distance between the vPCV and the bottom end of the SIV), was measured using ImageJ (National Institutes of Health).

### *In situ* hybridization

*Tg(EPV.Tp1-Mmu.Hbb:EGFP)* embryos were fixed in PBS containing 4% PFA. The EGFP-specific antisense riboprobe was transcribed *in vitro* from an ApaI-linearized 383.pME-EGFP plasmid (Tol2kit), using T7 RNA polymerase and a DIG labeling kit (Roche). Whole-mount *in situ* hybridization was performed according to Lauter et al. (2011). After flat mounting in 1% low-melting agarose, far-red emission from Fast Blue-stained embryos (Sigma-Aldrich) was acquired.

### Imaging

Confocal imaging was performed using a Zeiss LSM 780 upright confocal microscope (Carl Zeiss) with a W-Plan Apochromat 20× objective, NA 1.0. Whole-mount *in situ* hybridization images were acquired with a Leica SP5 spectral confocal microscope (633 nm laser line; 25× water dipping objective). Images of 12xNRE:EGFP embryos for EGFP intensity measurements were acquired using a Leica M165FC fluorescence microscope equipped with a Nikon DS-Fi2 camera. Images were processed using ImageJ (National Institutes of Health), Volocity (PerkinElmer) and Imaris (Bitplane). Fluorescent proteins were excited sequentially with 488 and 563 nm single-photon lasers.

Time-lapse *in vivo* imaging was performed as described by Nicenboim et al. (2015). *z*-stacks were acquired at 2.5-3 μm increments, every 9-16 min (shown in time stamp). For colocalization analyses, confocal images were analyzed using the Imaris ‘Co-localization Module’ and the Volocity 3D opacity utility. A new channel was applied to label with both EGFP and mCherry/dsRed fluorophores. Where necessary, movies were registered with the ‘Linear Stack Alignment with SIFT’ plugin of FIJI. For the Notch reporter *Tg(EPV.Tp1-Mmu.Hbb:EGFP)* validation, quantification of EGFP protein fluoresence and mRNA levels (detected via Fast Blue staining) was carried out using Volocity 6.0 software (Perkin Elmer). Quantification of EGFP florescence was performed on 50 μm×50 μm regions of interest (ROI) in the tail region, using the maximum pixel counting utility. Quantification of Fast Blue-stained fluorescence was performed on maximum projection files focusing on the hindbrain region (50 μm×50 μm ROI), using the mean pixel counting utility.

### Statistical analyses

Two-tailed Student's unpaired *t*-test assuming unequal variance from at least three independent experiments was used, unless stated otherwise. Numerical data are the mean±s.e.m., unless stated otherwise.
